# Differences in Chemical Sexual Signals May Promote Reproductive Isolation and Cryptic Speciation between Iberian Wall Lizard Populations

**DOI:** 10.1155/2012/698520

**Published:** 2012-01-11

**Authors:** Marianne Gabirot, Pilar López, José Martín

**Affiliations:** ^1^Départamento de Ecología Evolutiva, Museo Nacional de Ciencias Naturales, CSIC, José Gutiérrez Abascal 2, 28006 Madrid, Spain; ^2^Département Ecologie Comportementale, UMR 5175, CEFE-CNRS, 1919 route de Mende, 34293 Montpellier Cedex 5, France

## Abstract

Interpopulational variation in sexual signals may lead to premating reproductive isolation and speciation. Genetic and morphological studies suggest that the Iberian wall lizard, *Podarcis hispanica*, forms part of a “species complex” with several cryptic species. We explored the role of chemical sexual signals in interpopulational recognition between five distinct populations of Iberian wall lizards in Central Spain. Results showed that these populations differed in morphology and in composition and proportion of chemical compounds in femoral gland secretions of males. Tongue-flick experiments indicated that male and female lizards discriminated and were more interested in scents of lizards from their own area (i.e., Northern versus Southern populations), but did not discriminate between all populations. Moreover, only males from the populations that are geographically located more far away preferred scent of females from their own population. These data suggest that, at least between some populations, there may be reproductive isolation mediated by chemical signals and cryptic speciation.

## 1. Introduction

Interpopulational variation in sexual chemical signals may provide the basis for premating reproductive isolation and speciation in many animals [1, 2]. Phenotypic plasticity in sexual signals could play a key role in initial signal divergence [3], for example, as a way to maximize the efficiency of signals for communication in different environments [4, 5]. These differences can be later amplified by sexual selection leading to differences in mating preferences [6–8], which could preclude mating between populations (e.g., [9–13]), and lead to speciation processes.

In many lizards, intraspecific communication and sexual selection are based on chemical signals secreted by specific glands [14–17]. For example, chemical compounds secreted by femoral gland of males can convey information about social status [18–22] and genetic quality of a male [23–26]. Also, differences in chemical signals may preclude interspecific mating between related sympatric species (e.g., [27, 28]). We hypothesized that interpopulational variations in femoral gland secretions within the same species might lead to reproductive isolation and thus promote speciation processes.

The Iberian wall lizard, *Podarcis hispanica*, is a small diurnal lizard, living in rocky habitats of the Iberian Peninsula. Molecular and morphological studies suggest that this lizard is paraphyletic and forms part of a “species complex,” which suggests the existence of cryptic speciation within taxa previously considered to be conspecific [29–34]. Chemosensory recognition is well developed in *P. hispanica *[11, 35]. This lizard can discriminate between sexes by chemical cues alone [36–39]. Chemical cues of males, mainly from the femoral gland secretions, are important in male-male interactions [19, 20, 40] and in female mate choice decisions [35, 41, 42]. Also, chemical cues of females, in conjunction with coloration, elicit courtship by males [37]. At least two populations differ in chemical characteristics of femoral secretions of males [11]. This raises the possibility that *P. hispanica* lizards use chemical sexual signals to discriminate between populations, which might lead to reproductive isolation (if variation of signals is discrete or there is a barrier to gene flow) and explain the genetic and morphological differences observed between populations.

In this study, we explored the role of chemical sexual signals in interpopulational recognition between five distinct populations of Iberian wall lizards in Central Spain. In this area, several populations inhabiting different environments live close together without geographical barriers that isolate the populations, and individuals may find each other easily [11, 12, 43]. However, some populations maintain clear distinct morphotypes and differ genetically [32–34], which suggests that they might be, at least partly, reproductively isolated. We hypothesized that interpopulational variations in chemical signals could allow chemosensory recognition between populations and lead to premating boundaries. To test this, we first compared the morphological characteristics of these populations, and then we analyzed whether there was variation in the composition and proportions of chemical compounds in femoral gland secretions of males by using gas chromatography-mass spectrometry (GC-MS). We further conducted tongue-flick experiments to analyze whether males and females discriminated by chemosensory cues alone between scent of lizards from different populations. We hypothesized that male and female lizards could be able to recognize by chemical cues alone, and maybe prefer, the scents of individuals of their own population, which may contribute to a reduced gene flow. We expected that interpopulational differences in chemical signals of males and in population recognition abilities could suggest the probable existence of reproductive isolation and cryptic speciation between these Iberian wall lizard populations.

## 2. Methods

### 2.1. Study Populations

**Figure 1:**
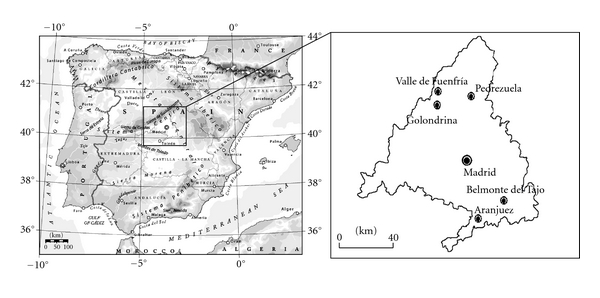
Geographic localization of the five populations of *Podarcis hispanica* studied in the Madrid region in the center of Spain.

During February-March 2008, we captured by noosing male and female *P. hispanica *lizards at five localities within the Madrid Region (Central Spain) (Figure 1). Three of these were localized in the Northern mountain area (“Fuenfría,” “Golondrina,” and “Pedrezuela”), and the other two were situated in the Southern plain area (“Belmonte” and “Aranjuez”). We selected these populations because lizards clearly differ in morphology and coloration [43, 44]. In the North, we captured lizards from a population occupying different granite rock cliffs at the edge of a pine forest in the upper part of the “Fuenfría” Valley (40°47′N, 04°03′W; 1750 m altitude; 21 males and 26 females), on granite rocky outcrops inside a large oak forest “Golondrina” near Cercedilla village (40°44′N, 04°02′W; 1250 m altitude; 29 males and 27 females), and from old stone walls near crop fields close to “Pedrezuela” village (40°44′N, 03°36′W; 800 m altitude; 19 males and 16 females). In the South, we captured lizards on human buildings and walls inside a public garden of the “Belmonte del Tajo” village (40°08′N, 03°20′W; 735 m altitude; 22 males and 17 females) and on chalk and gypsum rocks in deforested bushy hills near “Aranjuez” village (40°02′N, 03°37′W; 494 m altitude; 21 males and 32 females).

 All lizards were individually housed at “El Ventorrillo” Field Station (Cercedilla, Madrid) about 5 Km from the capture sites of the Northern populations, in indoor 60 × 40 cm PVC terraria containing sand substratum and rocks for cover. Cages were heated with 40 W spotlights during 6 h/day, and overhead lighted (36 W full-spectrum daylight tubes) on a 10 h : 14 h light/dark cycle, and were screened from each other using cardboard. Every day, lizards were fed mealworm larvae (*Tenebrio molitor*) dusted with multivitamin powder for reptiles, and water was provided *ad libitum*. Lizards were returned to their exact capture sites with good health condition at the end of experiments.

### 2.2. Morphological Characteristics

We made the following morphological measurements of each individual lizard: body mass (or weight) (measured with a digital balance to the nearest 0.01 g) and body size (snout-to-vent length, SVL; measured with a ruler to the nearest 1 mm). We also made morphological measurements of the head using a digital caliper (to the nearest 0.05 mm). Head length was the distance between the tip of the snout and the posterior side of the parietal scales. Head width was the greatest distance between the external sides of the parietal scales. Head depth was the greatest distance from the highest portion of the head to the bottom of the lower jaw.

 We also counted under a magnifying glass the number of femoral pores on the right and left hindlimbs of lizards and calculated an average number for both sides. Finally, we noted the number of small but distinctive and conspicuous blue ocelli that runs along each of the body sides on the outer margin of the belly of males and calculated an average number for both sides. These ocelli seem to have a role in sex recognition and intrasexual social relationships between males [39, 45].

All biometrical variables were log transformed prior to analysis to meet assumptions of normality and homoscedasticity. We used one-way analyses of variance (ANOVAs) to test for differences in morphological variables between populations. Pairwise comparisons were based on Tukey's honestly significant difference (HSD) tests [46].

### 2.3. Chemical Analyses of Femoral Gland Secretions

Immediately after capture in the field, we extracted femoral gland secretion of males by gently pressing with forceps around the femoral pores and collected secretion directly in glass vials with Teflon-lined stoppers. Vials were stored at −20°C until analyses. We also used the same procedure on each sampling occasion but without collecting secretion, to obtain blank control vials that were treated in the same manner to compare with the lizard samples. Before the analyses we added 250 *μ*L of *n*-hexane (Sigma, capillary GC grade) to each vial. We analyzed lipophilic compounds in samples by using a Finnigan-ThermoQuest Trace 2000 gas chromatograph (GC) fitted with a poly (5% diphenyl/95% dimethylsiloxane) column (Thermo Fisher, Trace TR-5, 30 m length × 0.25 mm ID, 0.25 mm film thickness) and a Finnigan-ThermoQuest Trace mass spectrometer (MS) as detector. Sample injections (2 *μ*L of each sample dissolved in *n*-hexane) were performed in splitless mode using helium as the carrier gas at 30 cm/sec, with injector temperature at 250°C. The oven temperature program was as follows: 50°C isothermal for 5 min, then increased to 270°C at a rate of 10°C/min, isothermal for 1 min, then increased to 315°C at rate of 15°C/min, and finally isothermal (315°C) for 10 min. Ionization by electron impact (70 eV) was carried out at 250°C. Mass spectral fragments below *m*/*z* = 39 were not recorded. Impurities identified in the solvent and/or the control vial samples are not reported.

Initial tentative identification of secretion components was done by comparison of mass spectra in the NIST/EPA/NIH 1998 computerized mass spectral library. Identifications were confirmed by comparison of spectra and retention times with those of authentic standards from Sigma-Aldrich Chemical Co. For unidentified or unconfirmed compounds we report here their characteristic ions, which we used together with retention times and characteristic *m*/*z* ratios to confirm whether these compounds were present in a given individual.

 For the statistical analyses of femoral secretions, the relative amount of each component was determined as the percent of the total ion current (TIC). The relative areas of the peaks were transformed following Aitchison's formula [47–49]. The homogeneity of variance of these variables was tested with Levene's test, and Bonferroni's correction was applied. The transformed areas were used as variables in a principal component analysis with varimax rotation. The eight principal components (PCs) extracted (all with eigenvalues >1, which explained 82.55% of variance) were used as covariates in a discriminant analysis to test whether chemical compounds in femoral secretions could be used to predict the population of origin of a male lizard. Then, we calculated the squared Mahalanobis distances of individuals with all other individuals and compared them between populations.

### 2.4. Chemosensory Recognition between Populations

Lizards have been shown to react to a variety of chemical stimuli with increased and differential rates of tongue extrusions [50]. Tongue-flick (TF) rate can, therefore, be used as a quantitative bioassay of detection of chemical cues (e.g., [11, 38]). Thus, to test for differential responses to scents, we made comparisons of TF rate by lizards (males and females) in response to chemical stimuli presented on cotton applicators impregnated with scents of male or female *P. hispanica *from each of the five different populations (Aranjuez, Golondrina, Fuenfría, Pedrezuela, and Belmonte) or with deionized water (odorless control). Water was used to gauge baseline TF's rates in the experimental situation [50]. We obtained lizard scents from the femoral pores of males or from the cloacal area of females because these are the body areas most frequently and intensely investigated by tongue flicking during social encounters [19, 37, 39]. Therefore, after first dipping the cotton tip (1 cm) of a wooden applicator attached to a long stick (50 cm) in deionized water, we rolled the tip over those body areas (of one population and sex per applicator). We used a new applicator in each trial.

 First, males were exposed to scents from males and then to scents from females of each population tested. Finally we studied the responses of females to scent of males of each population. Every lizard was exposed to each stimulus and order of presentation was counterbalanced. One trial was conducted per day for each animal. Trials were conducted in outdoor conditions during April, which coincided with the mating season of lizards in their original natural populations (P. López and J. Martín, unpublished data), and between 11:00 and 13:00  (GMT) when lizards were fully active.

 To begin a trial, the experimenter slowly approached the terrarium and slowly moved the cotton swab to a position 1 cm anterior to the lizards' snout. Lizards usually did not flee from the swab, but explore it repeatedly by tongue flicking or ignore it after the first TFs. In all cases, lizards directed TFs to the swab in all conditions. The numbers of TFs directed at the swab were recorded for 60 s beginning with the first TF. Analyses were made separately for responding males and females. To examine differences among treatments, previous analyses showed that responses to the different scents differed as a function of the population of the responding lizard. Thus, we used separated one-way repeated measures ANOVAs to test the effect of scent stimuli (within factor; Fuenfría versus Golondrina versus Aranjuez versus Pedrezuela versus Belmonte versus water) in number of TFs directed at the swab within each population of responding lizards. Data were log-transformed to ensure normality. Tests of homogeneity of variances (Levene's test) showed that in all cases variances were not significantly heterogeneous after transformation. Pairwise comparisons were planned using Tukey's honestly significant difference (HSD) tests [46].

## 3. Results

### 3.1. Interpopulational Differences in Morphology

There were significant differences between populations in all morphological measurements (Table 1). In general, lizards from Fuenfría and Golondrina populations were significantly heavier and longer and had greater heads than lizards from Aranjuez and Belmonte, which did not differ. Lizards from Pedrezuela were intermediate in size between the other populations (Table 1). However, when the effect of variation in body size between populations was removed, head size differences were significant only for head depth (ANOVA on residuals of head size with SVL, *P* = 0.005 for both sexes), but not for head length (*P* > 0.20 for both) or width (*P* > 0.05 for both). With respect to the number of femoral pores, both male and female lizards from Aranjuez had significantly less femoral pores than lizards from Belmonte, Fuenfría, and Golondrina, which did not differ. Lizards from Pedrezuela had an intermediate number of pores (Table 1). The number of femoral pores was not significantly related to body size (*P* > 0.60 in all cases). Finally, males from Aranjuez, Belmonte, and Pedrezuela had significantly more blue ocelli than males from Fuenfría and Golondrina (Table 1).

### 3.2. Interpopulational Differences in Chemical Composition of Femoral Secretions

**Figure 2:**
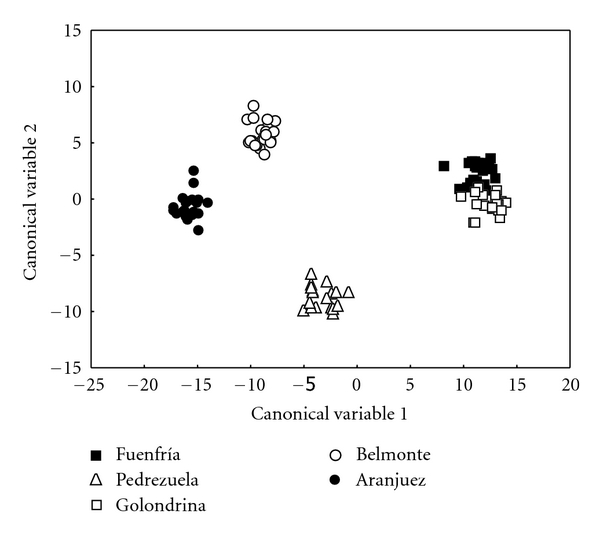
Separation of the principal components scores (PCs) describing chemicals from femoral secretions of male lizards in a discriminant analysis based on population of origin.

We found 53 lipophilic compounds in femoral gland secretions of male *P. hispanica* (Table 2). The lipophilic fraction of femoral gland secretions of males, all five populations pooled, is a mixture of steroids (83.69% of TIC), and carboxylic acids ranged between *n*-C_14_ and *n*-C_22_ and their esters (10.30%), but we found also five alcohols between *n*-C_16_ and *n*-C_24_ (3.53%), a furanone (1.18%), four waxy esters (1.10%), squalene (0.60%), and two terpenoids (0.28%). On average, the five most abundant chemicals were cholesterol (63.24% of TIC), followed by cholesta-5,7-dien-3-ol (5.16%), hexadecanoic acid (3.73%), campesterol (3.66%), octadecenoic acid (2.46%), and octadecanoic acid (1.77%). There were 34 chemical compounds shared by lizards from all populations, but we found differences between populations in the presence/absence of 19 compounds in femoral secretions (Table 2). The discriminant analysis showed that the eight PCs scores describing proportions of compounds in femoral secretions could be used to predict the population of origin of a male lizard (Wilks' *λ* = 0.0001, F_32,355_ = 607.45, *P* < 0.0001) (Figure 2). All the pairwise comparisons of the Mahalanobis distances between populations, which ranged between 150.35 and 1015.13, were significant in all cases (210.46 < F_8,96 _< 1290.20, *P* < 0.0001 in all cases).

### 3.3. Chemosensory Responses of Males to Scent of Males

**Figure 3:**
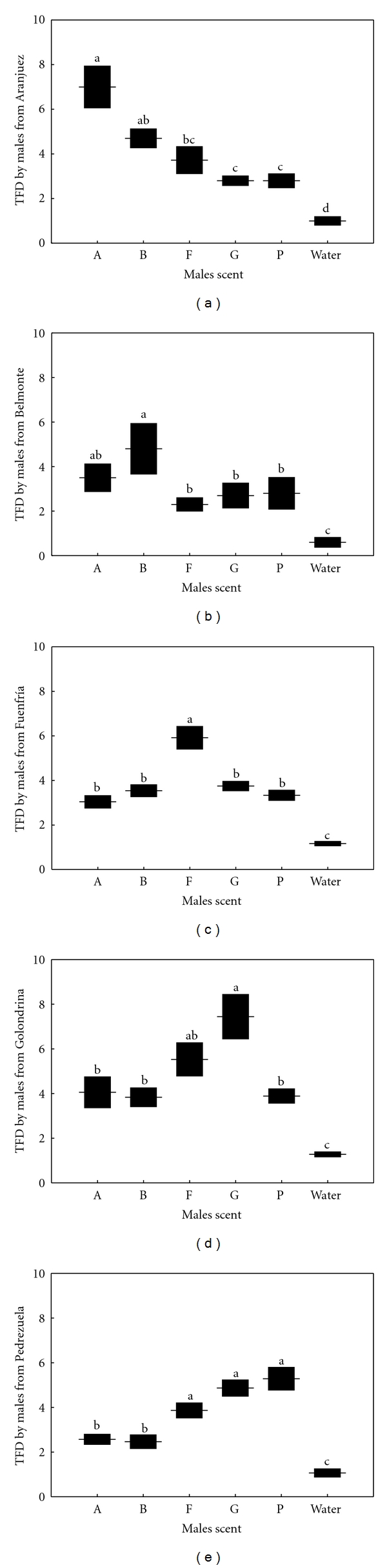
Tongue flicks directed (TFD; mean ± SE) by males from five populations of the Madrid region in response to swabs bearing scent of males of different populations (Aranjuez: A; Belmonte: B; Fuenfría: F; Golondrina: G; Pedrezuela: P) or a water odorless control. The same letter above the bars denotes a nonsignificant difference in post hoc tests.

The number of TFs differed significantly between the scents presented in all cases (Table 3; Figure 3). In all populations, males discriminated between scents of any male and water (Tukey's tests: *P* < 0.005 in all cases).   Males from Aranjuez and Belmonte directed a significantly higher number of TFs to scent of males of their own population or of the other Southern population than to scent of males from the three Northern populations, which did not differ (Table 3; Figures 3(a) and 3(b)). The number of TFs directed by males from Fuenfría was significantly higher in response to scent of males of their own population than to scent of males from any other population, which did not significantly differ (Table 3; Figure 3(c)). Males from Golondrina directed a significantly higher number of TFs in response to scent of males of their own population than to males from Aranjuez, Belmonte and Pedrezuela (Table 3; Figure 3(d)). The number of TFs in response to scent of males of their own population and Fuenfría males was not significantly different, and the latter was not significantly different from the rest of populations. Finally, males from Pedrezuela directed significantly more TFs in response to males of their own population than to males of the two Southern populations (Aranjuez and Belmonte), which did not significantly differ (Table 3; Figure 3(e)). However, responses to males of their own population did not significantly differ from responses to males of the other two Northern populations.

### 3.4. Chemosensory Responses of Males to Scent of Females

**Figure 4:**
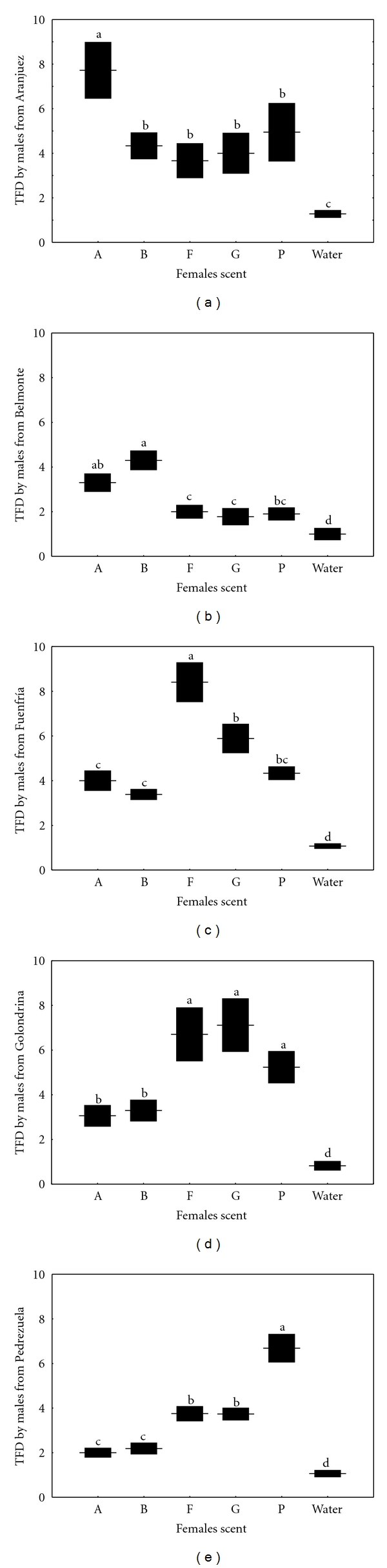
Tongue flicks directed (TFD; mean ± SE) by males from five populations of the Madrid region in response to swabs bearing scent of females of different populations (Aranjuez: A; Belmonte: B; Fuenfría: F; Golondrina: G; Pedrezuela: P) or a water odorless control. The same letter above the bars denotes a nonsignificant difference in post hoc tests.

The number of TFs differed between treatments in all populations (Table 3; Figure 4). In all cases, males discriminated between scents of any female and water (Tukey's tests: *P* < 0.005 in all cases). Males from Aranjuez and Belmonte directed a significantly higher number of TFs to scent of females of their own population than to scent of females from all the Northern populations, which did not significantly differ (Table 3; Figures 4(a) and 4(b)). The number of TFs directed by males from Fuenfría was significantly higher in response to scent of females of their own population than to females from any other population (Table 3; Figure 4(c)). Males from Golondrina directed a significantly higher number of TFs in response to scent of females from the three Northern populations, including their own population, than to females from the two Southern populations (Table 3; Figure 4(d)). Males from Pedrezuela directed significantly more TFs in response to scent of females of their own population than to scent of females from any other population (Table 3; Figure 4(e)). However, responses to scent of females from the two other Northern populations were significantly higher than to females from the two Southern populations, which did not differ.

### 3.5. Chemosensory Responses of Females to Scent of Males

**Figure 5:**
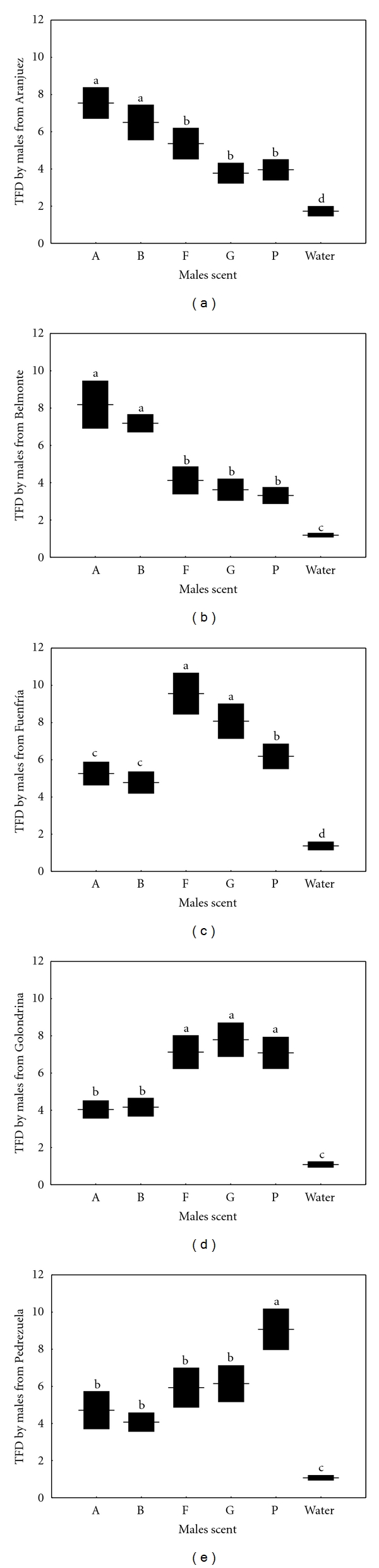
Tongue flicks directed (TFD; mean ± SE) by females from five populations of the Madrid region in response to swabs bearing scent of males of different populations (Aranjuez: A; Belmonte: B; Fuenfría: F; Golondrina: G; Pedrezuela: P) or a water odorless control. The same letter above the bars denotes a nonsignificant difference in post hoc tests.

The number of TFs differed between treatments in all populations (Table 3; Figure 5). All females discriminated between scents of any male and water (Tukey's tests: *P* < 0.005 in all cases). Females from Aranjuez and Belmonte directed a significantly higher number of TFs in response to scent of males of their own population than to males from the three Northern populations, which did not differ significantly (Table 3; Figures 5(a) and 5(b)). Females from Aranjuez and Belmonte did not significantly differ in their responses to scent of males of their own population or to males from the other Southern population (Belmonte or Aranjuez). The number of TFs directed by females from Fuenfría was significantly higher in response to scent of males of their own population than to males from the two Southern populations and from one of the Northern populations (Pedrezuela), which did not significantly differ (Table 3; Figure 5(c)). Responses to scent of males of their own population and to males from Golondrina were not significantly different, nor were different the responses to males from Golondrina and Pedrezuela. Females from Golondrina directed a significantly higher number of TFs in response to scent of males from the three Northern populations (Fuenfría, Pedrezuela, and their own population) than to scent of males from the Southern populations (Aranjuez and Belmonte) (Table 3; Figure 5(d)). Females from Pedrezuela directed significantly more TFs in response to scent of males from their own population than to males from all the other Southern and Northern populations, which did not differ significantly (Table 3; Figure 5(e)).

## 4. Discussion

Our study showed that different populations of Iberian wall lizards *P*. *hispanica* living within a relatively small geographical area, whose environmental conditions differed between population sites, differed in morphology and in the composition and proportion of chemical compounds in femoral gland secretions of males. Males of each population secreted a singular and characteristic mixture of compounds used as sexual signals. Tongue-flick tests showed that these differences resulted in differential chemosensory recognition between some populations. These results suggested that there could be premating reproductive isolation between some, but not all, populations of this lizard.

 With respect to morphology, we could first differentiate between individuals from the South and North of the study area. Lizards from Fuenfría and Golondrina (i.e., Northern populations) were characterized by being larger, heavier, and with larger, more robust heads than individuals from Aranjuez and Belmonte (i.e., Southern populations). These differences could be explained by the different contrasting environments where these populations live, Northern mountains (with cold temperature, high humidity, and high altitude) versus Southern plains (hot temperatures, dry conditions, and low altitude). Variations of body size of many animals, and in particular of vertebrates, are often explained by phenotypic plasticity or local adaptation to different climatic conditions, with individuals from colder environments being larger than those from warmer areas (e.g., [51]). Lizards with a large body size have low thermal inertia (i.e., low cooling rates) [52], and this may be an adaptation to increase effectiveness of thermoregulation in the Northern populations where ambient temperatures are relatively cold, in contrast to the Southern populations where temperatures are warmer and lizards are smaller.

Moreover, Iberian wall lizard populations differ in the number of femoral pores and blue spots, with males from the Northern populations having more femoral pores and less blue spots than males from the Southern populations. Only lizards from the Pedrezuela Northern population had an intermediate number of femoral pores. Because femoral pores and blue spots are used in chemical and visual intraspecific communication, respectively (e.g., [37, 39]), it is likely that the importance of these two sensory modes differ between populations. A higher number of femoral pores may be related to a higher production of chemical secretions [53], whereas a larger number of blue spots may represent a higher use of visual signals [45]. The relative importance of chemical and visual signals may be explained by the effectiveness of these two types of communication in different environments [4, 54], which might have affected the evolution of sexual signals of different populations of *P. hispanica* lizards.

 In fact, the chemical analyses showed that, similarly to other lizard species, femoral gland secretions of *P. hispanica* have carboxylic acids and steroids as predominant components (reviewed in [55]). However, compounds found in femoral gland secretions of male *P*. *hispanica *varied in composition and proportions between populations, and these variations alone would allow a characterization of males from each population. These differences could be due to local adaptation to the habitats of each population [4, 54]. Selection for a better efficiency of substrate scent marks might have led to differences in composition of secretions of lizards inhabiting distinct environments, with less volatile and stable molecules being found in the Southern populations where temperature and evaporation rates were higher [4, 11]. Also, differences in secretions might be related to different diets or differently available food sources [5]. The question that arises is whether these differences in chemicals affect recognition systems and whether this may have consequences for speciation.

Chemosensory recognition experiments showed that individuals of *P. hispanica* from each population could clearly detect scents of lizards from any population in comparison with an odorless control (i.e., water). However, lizards showed different tongue-flick (TF) rates depending on the population of origin of the lizard's scent presented. Both females and males varied in their responses to scents from lizards from the different populations. Males showed more “interest” (i.e., a higher TF rate) for scents from males from their own area (i.e., North versus South); males from the Northern populations made more TFs in response to scent of males from the Northern populations than to scent of Southern males. Similarly, Southern males made more TFs in response to scents from Southern males than to scent from Northern males. Only males from Fuenfría population showed a clearly higher response to scents from males of their own population. For the rest of populations, there were not higher responses to scent of males from their own population, but there was a recognition of the area of origin (North versus South) of the male.

Moreover, males also discriminated between scents from females from the different areas. Males from Northern populations showed more interest for scents from Northern females than for scents from Southern females; similarly this occurred in Southern males. However, we observed one interesting difference: males from the populations that are geographically located far away from the others (i.e., Aranjuez, Fuenfría, and Pedrezuela) showed a clear discrimination and interest (i.e., higher TF rates) for scents of females from their own population against scent of females from any other population. There was also a further secondary intermediate interest for scent of females from other populations of their own area and finally a lower interest for females from the other area. In contrast, for the populations geographically located in the middle of the Madrid region (i.e., Belmonte and Golondrina), we did not observe a discrimination nor a higher interest of males for scent of females from their own population, but only a discrimination of females from their own area.

In addition, we found similar results for the males' scents recognition by females. Females recognized the area of origin of the male (South versus North). Females from Northern populations made more TFs in response to scent of Northern males than to Southern males, and vice versa, but there were no differences between populations within each area. We found only a clearly higher interest of Pedrezuela females for scents of males from their own population against all the other populations.

These results seem concordant with the previous description of morphotypes of *P. hispanica* using morphological and genetic data [29, 32–34]. Thus, Northern populations would be close to those described for the morphotype 1, while the Southern populations would be more similar to the morphotype 2. However, we observed a particular result for lizards from Pedrezuela population; these lizards live in the North, but they have chemical and morphological differences with respect to other Northern populations. Lizards from Pedrezuela have a morphology intermediate between Southern and Northern populations. Moreover, the chemical signals in this population are singular in comparison to the other populations, and this chemical signature is effective in the chemosensory recognition of scent of males and female from their own population. Therefore, the assignation of this population to previously described morphotypes is not clear.

In summary, our results showed that male and female *P. hispanica* lizards from five distinct populations of the Madrid region can recognize and discriminate between scents of individuals from the Northern and Southern populations, and have more interest for scents of lizards from their own area than for scents of lizards from the other area. Moreover, males from some populations discriminate and maybe prefer scents of females from their own population than from any other populations. This clear ability of males to discriminate between some female populations might suggest that there is a cryptic speciation process, probably mediated by the role of chemical signals in sexual interactions. However, we need further mating experiments to test this. In addition, females also seem to discriminate male chemicals between areas (North versus South), but not between populations. All these results support that reproductive isolation between all the distinct populations of *P. hispanica* is not entirely clear, but that, at least between some populations, there could be reproductive isolation and cryptic speciation, which merits further studies.

## Figures and Tables

**Table 1:** Morphological characteristics (mean ± SE) of *P. hispanica* lizards (males and females) from five distinct populations of the Madrid region (Aranjuez, Belmonte, Golondrina, Fuenfría, and Pedrezuela). Results from one-way ANOVAs comparing morphological measures between populations are shown. The same letter after the means denotes a nonsignificant difference in post hoc tests.

Morphological measures	Populations					ANOVAs	
	Aranjuez	Belmonte	Golondrina	Fuenfría	Pedrezuela	F_4,107_	*P*
*Males*							
Weight (g)	3.3 ± 0.2^ab^	3.1 ± 0.2^a^	4.7 ± 0.2^bc^	5.5 ± 0.2^c^	3.8 ± 0.2^b^	35.89	< 0.001
SVL (mm)	51 ± 1^ab^	50 ± 1^a^	59 ± 1^bc^	62 ± 1^c^	55 ± 1^b^	38.80	< 0.001
Head length (mm)	12.7 ± 0.2^a^	12.4 ± 0.2^ac^	14.2 ± 0.2^bc^	14.9 ± 0.1^b^	13.8 ± 0.2^c^	16.57	< 0.001
Head width (mm)	7.3 ± 0.1^a^	7.5 ± 0.1^ac^	8.2 ± 0.1^bc^	8.5 ± 0.1^b^	8.0 ± 0.1^c^	11.96	<0.001
Head depth (mm)	5.3 ± 0.1^a^	5.6 ± 0.1^ac^	6.1 ± 0.1^bc^	6.1 ± 0.1^b^	5.9 ± 0.1^c^	31.20	<0.001
Femoral pores	16.2 ± 0.2^a^	17.5 ± 0.3^b^	18.3 ± 0.3^b^	17.7 ± 0.3^b^	17.2 ± 0.3^ab^	7.12	<0.001
Blue ocelli	4.5 ± 0.5^a^	4.9 ± 0.5^a^	1.9 ± 0.5^b^	1.7 ± 0.4^b^	5.9 ± 0.6^a^	13.41	<0.001

*Females*						F_4,113_	*P*
Weight (g)	2.9 ± 0.1^ab^	2.5 ± 0.1^a^	2.9 ± 0.1^ab^	3.3 ± 0.2^b^	2.8 ± 0.2^ab^	3.20	0.015
SVL (mm)	50 ± 1^ab^	50 ± 1^ab^	55 ± 1^bc^	56 ± 1^c^	52 ± 1^b^	11.43	<0.001
Head length (mm)	11.0 ± 0.1^a^	11.1 ± 0.2^a^	11.8 ± 0.1^b^	12.0±0.1^b^	11.5 ± 0.2^a^	3.49	0.01
Head width (mm)	6.6 ± 0.1^a^	6.5 ± 0.1^a^	6.7 ± 0.1^b^	6.9 ± 0.1^b^	6.6 ± 0.1^a^	3.48	0.01
Head depth (mm)	4.8 ± 0.1^a^	4.8 ± 0.1^a^	5.0 ± 0.1^b^	5.1 ± 0.1^b^	4.7 ± 0.1^a^	9.40	<0.001
Femoral pores	13.7 ± 0.2^a^	16.4 ± 0.3^b^	15.6 ± 0.2^b^	16.0 ± 0.2^b^	15.3 ± 0.2^ab^	21.62	<0.001

**Table 2:** Lipophilic compounds found in femoral gland secretions of male lizards, *P. hispanica*, from five distinct populations of the Madrid region (Aranjuez, Belmonte, Golondrina, Fuenfría, and Pedrezuela). The relative amount of each component was determined as the percent of the total ion current (TIC) and reported as the average (±SE). Characteristics (*m*/*z*) are reported for some unidentified (Un.) compounds. (RT: retention time).

Compounds	RT (min)	Fuenfría	Pedrezuela	Golondrina	Belmonte	Aranjuez
*Steroids*						
Un. steroid (145,213,248,353,368,387)	29.92	0.01 ± 0.01	—	0.17 ± 0.05	1.49 ± 0.56	—
Cholesta-2-4-diene	30.58	0.68 ± 0.11	2.66 ± 0.44	0.44 ± 0.08	2.59 ± 0.46	0.96 ± 0.35
Cholesta-3.5-diene	30.81	0.42 ± 0.10	0.23 ± 0.04	0.30 ± 0.07	0.13 ± 0.03	0.25 ± 0.07
Un. steroid (155,197,251,350,365)	30.96	1.32 ± 0.16	1.00 ± 0.14	0.55 ± 0	0.45 ± 0.06	0.45 ± 0.17
Cholesta-5,7,9(11)-trien-3-ol	31.06	1.62 ± 0.18	1.07 ± 0.24	0.94 ± 0.11	0.65 ± 0.11	0.29 ± 0.07
Un. steroid (207,251,350,365)	31.13	0.40 ± 0.08	0.16 ± 0.02	0.18 ± 0.02	0.18 ± 0.07	0.08 ± 0.04
Un. steroid (143,195,207,351,366)	31.20	0.19 ± 0.02	0.08 ± 0.01	0.15 ± 0.04	0.18 ± 0.05	0.22 ± 0.06
Un. steroid (141,156,209,350,365)	31.37	0.37 ± 0.05	0.03 ± 0.01	0.30 ± 0.06	2.47 ± 0.42	—
Un. steroid (155,197,251,365,379)	31.64	0.06 ± 0.01	0.21 ± 0.02	0.08 ± 0.02	0.43 ± 0.07	0.45 ± 0.18
Un. steroid (195,209,251,365,379)	31.84	—	0.07 ± 0.01	0.27 ± 0.07	0.51 ± 0.08	0.32 ± 0.12
Cholesterol	32.43	59.74 ± 2.79	62.33 ± 1.68	66.61 ± 2.00	53.03 ± 2.51	74.51 ± 2.04
Cholestanol	32.47	1.40 ± 0.14	0.53 ± 0.08	0.90 ± 0.11	0.60 ± 0.06	0.55 ± 0.12
Cholesta-5.7-dien-3-ol.	32.65	13.41 ± 1.85	2.68 ± 0.54	8.02 ± 1.33	1.16 ± 0.19	0.54 ± 0.17
Un.steroid (105,213,255,353,368,386,415)	32.75	0.02 ± 0.01	0.03 ± 0.02	0.35 ± 0.11	0.09 ± 0.03	0.39 ± 0.16
Ergosterol	33.00	—	0.05 ± 0.02	—	0.17 ± 0.11	—
Campesterol	33.17	1.61 ± 0.22	3.76 ± 0.28	3.27 ± 0.36	5.46 ± 0.28	4.22 ± 0.57
Cholest-4-en-3-one	33.41	0.17 ± 0.03	0.53 ± 0.17	0.19 ± 0.05	0.20 ± 0.02	0.92 ± 0.38
Ergosta-5,8-dien-3-ol	33.50	2.43 ± 0.30	1.58 ± 0.22	2.38 ± 0.37	1.31 ± 0.24	0.56 ± 0.14
Cholesta-4,6-dien-3-one	33.69	0.24 ± 0.06	0.53 ± 0.08	0.29 ± 0.06	0.40 ± 0.06	—
Sitosterol	33.92	0.65 ± 0.10	0.74 ± 0.16	0.94 ± 0.15	1.18 ± 0.11	1.13 ± 0.23
Ergostanol	34.02	0.07 ± 0.01	0.08 ± 0.03	0.10 ± 0.02	0.11 ± 0.02	0.33 ± 0.11
Stigmasterol	34.13	0.31 ± 0.06	0.27 ± 0.13	0.28 ± 0.04	1.22 ± 0.22	0.44 ± 0.26
Un.steroid (221,253,281,355,380,430)	34.30	2.23 ± 0.32	0.70 ± 0.18	1.01 ± 0.16	—	—
Cholest-5-en-3-one	34.38	—	—	—	1.33 ± 0.24	0.91 ± 0.28
Ergosta-5.22-dien-3-ol	34.47	—	0.13 ± 0.07	0.12 ± 0.03	0.15 ± 0.04	—
Un.steroid (214,267,395)	35.30	0.12 ± 0.04	0.21 ± 0.11	—	0.56 ± 0.44	0.22 ± 0.09

*Carboxylic acids and their esters*						
Tetradecanoic acid	20.64	0.16 ± 0.04	0.38 ± 0.13	0.22 ± 0.06	0.24 ± 0.05	0.85 ± 0.55
Pentadecanoic acid	21.68	0.13 ± 0.02	0.15 ± 0.12	0.10 ± 0.03	0.18 ± 0.05	0.41 ± 0.19
Hexadecanoic acid. methyl ester	22.33	—	0.05 ± 0.02	—	0.09 ± 0.02	0.25 ± 0.08
Hexadecenoic acid	22.54	0.16 ± 0.02	0.40 ± 0.20	0.25 ± 0.07	0.57 ± 0.33	0.28 ± 0.09
Hexadecanoic acid	22.76	3.68 ± 0.32	4.36 ± 0.65	3.11 ± 0.35	5.98 ± 0.51	1.54 ± 0.23
Hexadecanoic acid, ethyl ester	22.98	—	0.37 ± 0.11	—	0.19 ± 0.06	0.40 ± 0.17
9,12-octadecadienoic acid	24.35	0.10 ± 0.01	0.11 ± 0.02	0.12 ± 0.02	0.27 ± 0.08	0.06 ± 0.02
Octadecenoic acid	24.43	1.99 ± 0.18	1.76 ± 0.20	2.76 ± 0.57	4.82 ± 1.41	1.01 ± 0.21
Octadecanoic acid	24.60	1.39 ± 0.12	2.52 ± 0.34	1.41 ± 0.13	2.55 ± 0.23	0.99 ± 0.18
Octadecanoic acid, ethyl ester	24.82	—	0.51 ± 0.23	—	0.14 ± 0.04	0.55 ± 0.23
Eicosanoic Acid	26.31	0.46 ± 0.09	0.63 ± 0.15	0.76 ± 0.11	0.59 ± 0.17	0.64 ± 0.18
Docosanoic acid	28.00	—	0.01 ± 0.01	—	0.01 ± 0.01	—
Docosanoic acid, ethyl ester	28.21	—	0.45 ± 0.12	—	0.21 ± 0.05	0.23 ± 0.12

*Alcohols*						
Hexadecanol	21.02	0.23 ± 0.05	—	0.19 ± 0.07	0.16 ± 0.04	0.16 ± 0.05
Octadecanol	23.87	0.26 ± 0.05	0.69 ± 0.16	0.19 ± 0.06	0.29 ± 0.08	—
Eicosanol	25.67	0.17 ± 0.03	0.55 ± 0.13	0.28 ± 0.08	0.21 ± 0.05	0.81 ± 0.28
Docosanol	27.33	0.23 ± 0.05	0.52 ± 0.15	0.23 ± 0.04	0.23 ± 0.04	0.73 ± 0.26
Tetracosanol	29.80	0.03 ± 0.01	0.07 ± 0.01	0.02 ± 0.01	0.07 ± 0.02	0.01 ± 0.01

*Waxy esters*						
Unidentified waxy ester 1	29.45	0.28 ± 0.08	0.98 ± 0.20	—	1.37 ± 0.47	0.75 ± 0.23
Unidentified waxy ester 2	35.57	0.58 ± 0.10	2.61 ± 0.60	0.42 ± 0.08	2.84 ± 0.45	0.69 ± 0.26
Unidentified waxy ester 3	38.06	0.23 ± 0.06	0.29 ± 0.06	0.20 ± 0.05	0.09 ± 0.03	0.37 ± 0.23
Unidentified waxy ester 4	38.27	0.63 ± 0.11	1.78 ± 0.26	0.47 ± 0.10	2.26 ± 0.30	0.82 ± 0.16

*Others*						
Tetradecanone	22.11	0.20 ± 0.05	0.27 ± 0.11	0.13 ± 0.03	0.15 ± 0.03	0.18 ± 0.06
Unidentified Furanone	24.19	0.12 ± 0.02	0.10 ± 0.03	0.06 ± 0.01	—	—
Squalene	30.07	0.93 ± 0.26	0.70 ± 0.10	0.66 ± 0.20	0.35 ± 0.04	0.40 ± 0.19
Unidentified terpenoid 1	30.83	0.09 ± 0.03	0.07 ± 0.03	0.08 ± 0.02	0.03 ± 0.01	0.13 ± 0.07
Unidentified terpenoid 2	31.94	0.48 ± 0.09	—	0.48 ± 0.12	0.05 ± 0.01	—

**Table 3:** Results from one-way repeated measures ANOVAs comparing the tongue flicks directed by individuals (males and females) from five distinct populations of the Madrid region (Aranjuez, Belmonte, Golondrina, Fuenfría, and Pedrezuela) in response to swabs bearing scent of males or females of the different populations or a water odorless control.

	One-way repeated measures ANOVAs					
	*Responses of males to scent of males*		*Responses of males to scent of females*		*Responses of females to scent of males *	
	F	*P*	F	*P*	F	*P*
Aranjuez	36.46	<0.0001	24.39	<0.0001	27.30	<0.0001
Belmonte	35.98	<0.0001	21.89	<0.0001	33.67	<0.0001
Fuenfría	54.42	<0.0001	74.84	<0.0001	46.84	<0.0001
Golondrina	32.00	<0.0001	28.00	<0.0001	49.21	<0.0001
Pedrezuela	39.19	<0.0001	49.48	<0.0001	33.12	<0.0001
